# Study of the differentially abundant proteins among *Leishmania amazonensis*, *L*. *braziliensis*, and *L*. *infantum*

**DOI:** 10.1371/journal.pone.0240612

**Published:** 2020-10-15

**Authors:** Bruna Soares de Souza Lima, Barbara Beiral Esteves, Luiz Carlos Fialho-Júnior, Tiago Antônio de Oliveira Mendes, Simone da Fonseca Pires, Alexander Chapeourouge, Jonas Perales, Helida Monteiro de Andrade

**Affiliations:** 1 Departamento de Medicina, Faculdade Dinâmica do Vale do Piranga (FADIP), Ponte Nova, Minas Gerais, Brazil; 2 Departamento de Parasitologia, Laboratório de Leishmanioses, Instituto de Ciências Biológicas, Universidade Federal de Minas Gerais, Belo Horizonte, Minas Gerais, Brazil; 3 Departamento de Bioquímica e Biologia Molecular, Universidade Federal de Viçosa (UFV), Viçosa, Minas Gerais, Brazil; 4 Laboratório de Toxinologia, Instituto Oswaldo Cruz, Fiocruz, Rio de Janeiro, Brazil; Beni Suef University, Faculty of Veterinary Medicine, EGYPT

## Abstract

Leishmaniasis has been considered as emerging and re-emerging disease, and its increasing global incidence has raised concerns. The great clinical diversity of the disease is mainly determined by the species. In several American countries, tegumentary leishmaniasis (TL) is associated with both *Leishmania amazonensis* and *L*. *braziliensis*, while visceral leishmaniasis (VL) is associated with *L*. *(L*.*) infantum*. The major molecules that determine the most diverse biological variations are proteins. In the present study, through a DIGE approach, we identified differentially abundant proteins among the species mentioned above. We observed a variety of proteins with differential abundance among the studied species; and the biological networks predicted for each species showed that many of these proteins interacted with each other. The prominent proteins included the heat shock proteins (HSPs) and the protein network involved in oxide reduction process in *L*. *amazonensis*, the protein network of ribosomes in *L*. *braziliensis*, and the proteins involved in energy metabolism in *L*. *infantum*. The important proteins, as revealed by the PPI network results, enrichment categories, and exclusive proteins analysis, were arginase, HSPs, and trypanothione reductase in *L*. *amazonensis*; enolase, peroxidoxin, and tryparedoxin1 in *L*. *braziliensis*; and succinyl-CoA ligase [GDP -forming] beta-chain and transaldolase in *L*. *infantum*.

## Introduction

Leishmaniasis is endemic in 98 countries, and it is estimated that globally approximately 0.2 million to 0.4 million cases of visceral leishmaniasis and 0.7 million to 1.2 million cases of cutaneous leishmaniasis occur per year. India, Bangladesh, Sudan, Brazil, and Ethiopia accumulate more than 90% of the visceral leishmaniasis cases; and Afghanistan, Algeria, Colombia, Brazil, Iran, Syria, Ethiopia, Sudan, Costa Rica, and Peru contribute to approximately 75% of the cutaneous leishmaniasis cases [1].

Approximately 20 *Leishmania* species have been known to cause cutaneous or visceral infections in humans [2]. In the American continent, *Leishmania amazonensis*, *L*. *braziliensis*, and *L*. *infantum* are associated with a great clinical diversity. *L*. *amazonensis* is associated with diffuse (DCL) and localized cutaneous leishmaniasis (CL) [3], *L*. *braziliensis* is associated with localized cutaneous (CL) and mucocutaneous leishmaniasis (MCL) [4], whereas *L*. *infantum* is responsible for the visceral form (VL) of the disease [1].

CL is characterized by chronic papular, erythematous, and/or ulcerative skin lesions. MCL involves the destruction of the mucocutaneous tissues such as the nose, the nasal septum, the oropharynx, and the palate tissues, therefore, is associated with a high morbidity [5]. DCL is characterized by the presence of nodules and, in some cases, ulcerated lesions. In addition, histopathological examination has shown that macrophages are intensely parasitized in DCL, therefore, treatment is frequently ineffective. VL is the most serious form of the disease due to systemic involvement and infection with viscerotropic *L*. *infantum* strains. The disease manifests with persistent fever, enlargement of the liver and spleen, and pancytopenia, which is fatal if left untreated. This broad clinical spectrum results from the complex interactions between pathogenic virulence factors and the host immune system [6, 7].

Comparative analysis of the trypanosomatid genomes has revealed a high degree of syntenia, approximately 99% among *Leishmania* species [8–10]. Thus, proteomics represents an aggregate of techniques that allows us to understand the cell based on the identification and quantification of proteins under different conditions, with two-dimensional electrophoresis (2-DE) being one of the important tools used for large scale study of proteins [11].

Proteomic studies in *Leishmania* were performed mainly to compare the different stages of the life cycles of *L*. *donovani* [12], *L*. *infantum* [13, 14], and *L*. *mexicana* [15]. The differences in the abundance of proteins among *L*. *infantum* strains [16, 17] and *L*. *amazonensis* strains [18] have also been described. In addition, comparative studies were performed between the species *L*. (*V*.) *guyanensis* and *L*. (*V*.) *panamensis* [19], and *L*. *amazonensis* and *L*. *major* [20]. These studies contribute to the understanding of several biological mechanisms of the parasite associated with infection, survival, pathogenesis, as well as drug resistance.

Previously, we used a proteomic approach coupled with an *in silico* analysis and identified the most abundant and immunogenic proteins in *L*. *amazonensis*, *L*. *braziliensis*,and *L*. *infantum* in order to improve the serological diagnostic tests for the tegumentar form [21]. In the present study, we focused on the differentially abundant proteins among the above-mentioned species. Future investigation of these proteins will enhance our understanding of the biological differences among these species and the probable association of these proteins with clinical forms.

## Materials and methods

### Parasites

*Leishmania amazonensis* (IFLA/BR/1967/PH8), *L*. *braziliensis* (MHOM/ BR/1975/M2904), and *L*. *infantum* (MHOM/BR/1972/BH46) promastigotes were grown at 23°C in Schneider’s medium (Gibco BRL, UK) supplemented with 10% heat-inactivated fetal bovine serum (Sigma, MO, USA), 200 U of penicillin per mL (Sigma, MO, USA), and 100 μg of streptomycin per mL (Sigma, MO, USA) at pH 7.4. Growth curve analysis revealed that all the species reached the logarithmic phase at day 4. The logarithmic phase promastigotes were centrifuged at 2000 *g* for 20 min at 4°C, and the pellet was collected and stored at −80°C for protein extraction. Three independently growing cultures of each *Leishmania* species were obtained (biological replicates).

### Protein extracts

The promastigote pellets were resuspended in a lysis buffer containing 8 M urea, 2 M thiourea, 4% CHAPS, 65 mM DTT, 40 mM Tris base, and a protease inhibitor mix (GE Healthcare, San Francisco, CA), in the proportion of 500 μL of the lysis buffer/10^9^ parasites. After 2 h of shaking at room temperature, the cell lysate was centrifuged at 10,000 *g* for 30 min, and the soluble fraction was stored at −80°C until use. The protein content was measured using a 2D-Quant kit (GE Healthcare, USA) following the manufacturer’s instructions.

### Experimental design

First, three representative Coomassie 2D gels from each independent biological replicate of *Leishmania* species (*L*. *amazonensis*, *L*. *braziliensis*, and *L*. *infantum*) were analyzed to evaluate the coefficient of covariation for each biological replicate. A pool of three biological replicates of each *Leishmania* species was used for analysis by DIGE. Five DIGE analyses were performed and, in each gel, we included samples from two *Leishmania* species stained with different dyes (Cy3 and Cy5) and an internal standard of the three species stained with Cy2. High intensity spots from each *Leishmania* species sample (*p*-value < 0.01) were selected for identification by mass spectrometry (MS).

### DIGE

In order to perform DIGE, 50 μg of the extracted proteins from each species (*L*. *amazonensis*, *L*. *braziliensis*, and *L*. *infantum*) was labeled with 400 pmol of N-hydroxysuccinimidyl-ester-derivatives of the cyanine dyes Cy2, Cy3, and Cy5 (GE Healthcare, USA) following the manufacturer’s instructions. The reaction was quenched by incubating with 1 mL of 10 mM lysine for 10 min on ice in the dark. A mixture of protein extracts from the three *Leishmania* species was labeled with Cy2 as an internal standard. In each gel, protein extract of one species was labeled with Cy3 and that of the other species was labeled with Cy5. The experiments were performed in triplicate, and a dye-swap was performed for all species.

Differentially-labeled extracts were pooled, reduced with 2% DTT, complemented with 2% ampholytes (pH 4–7) and adjusted to a final volume of 350 μL with a sample buffer (8 M urea, 2 M thiourea, and 4% CHAPS). Samples were then loaded onto IPG strips (18 cm, pH 4–7; GE Healthcare, USA) by overnight passive rehydration at room temperature. Rehydrated IPG strips were subjected to isoelectric focusing (IEF) in which the voltage was increased gradually to 8000 V and the maximum current was 50 μA/strip; electrophoresis was run for 60,000 Vh on an Ettan IPGphor system (GE Healthcare, USA) at 20°C. Focused IPG strips were equilibrated for 15 min in equilibration buffer (50 mM Tris-HCl pH 8.8, 6 M urea, 30% glycerol, 2% SDS, 0.002% bromophenol blue, and 125 mM DTT) and then alkylated for further 15 min in equilibration buffer containing 13.5 mM iodoacetamide. The strips were then subjected to 12% sodium dodecyl sulphate-polyacrylamide gel electrophoresis (SDS-PAGE) within low-fluorescence glass plates (GE Healthcare, USA), and 2D-gel electrophoresis was performed at 10°C using a current of 25 mA/gel for 30 min, followed by 50 mA/gel, with an Ettan DALT 6 unit (GE Healthcare, USA). Electrophoresis was performed in the dark using a Tris/glycine/SDS buffer.

Gels were scanned using a Typhoon Trio laser imager (GE Healthcare, USA) with excitation/emission wavelengths specific for Cy2 (488 nm/520 nm), Cy3 (532 nm/580 nm), and Cy5 (633 nm/670 nm). Images were analyzed using the DeCyder 2D software version 7.0 (GE Healthcare, USA). The t-test with false discovery rate correction was used for statistical analyses, and α < 0.05 was adopted as the level of significance. Protein spots that showed high abundance in each *Leishmania* species (p-value < 0.01) were selected for an MS identification. To manually remove the selected spots, the DIGE gels were also stained with colloidal CBB G-250 following procedures described previously [22].

### Identification of proteins by MS

Spots with differential abundance were manually excised, trypsinized, and desalted using Zip-Tips (C18 resin; P10, Millipore Corporation, Bedford, MA) as per a previously described [23]. Approximately 0.5 μL of the sample solution was mixed with 0.25 μL of the saturated matrix solution [10 mg/mL α-cyano-4-hydroxycinnamic acid (Aldrich, Milwaukee, WI) in 50% acetonitrile/0.1% trifluoroacetic acid]. Tryptic peptides were analyzed with a MALDI-ToF-ToF AB Sciex 5800 (AB Sciex, Foster City, CA) mass spectrometer. MS and MS/MS spectra were acquired in reflector mode to ensure optimal mass accuracy and peak resolution. Usually up to 15 most intense ion signals with signal-to-noise ratios above 2 were selected as precursors for the MS/MS acquisition. During this data-dependent analysis, an exclusion list with common trypsin autolysis masses and keratin masses was used. External calibration in MS mode was performed using a mixture of five peptides: des-Arg1- Bradykinin (m/z = 904.468), angiotensin I (m/z = 1296.685), Glu1-fibrinopeptide B (m/z = 1570.677), ACTH (18–39 clip) (m/z = 2465.199), and ACTH (7–38 clip) (m/z = 3657.929). Similarly, tandem mass spectra were externally calibrated using known fragment ion masses observed in the MS/MS spectrum of Glu1-fibrinopeptide B.

Peaklists were created using the “peaks to mascot” tool in the Explorer software of the AB Sciex 5800 mass spectrometer. Common settings were signal-to-noise ratio of 2 and minimum peak area of 10. Database searches were performed against an in-house created “*Leishmania*” (103,645 sequences) database. The following search parameters were used: no restrictions on the protein molecular weight; tryptic cleavage products allowing two tryptic missed cleavages; variable modifications of cysteine (carbamidomethylation), methionine (oxidation), asparagine and glutamine (deamidation); and pyroglutamate formation at the N-terminal glutamine of peptides. Decoy analysis revealed a false discovery rate of 0.8% considering the peptide identity. A second database search against all entries (32,611,672 sequences) of the NCBI-non-redundant database (www.ncbi.nlm.nih.gov/index.html) revealed nearly the same results and did not show losses in the sensitivity of protein identification. The mass tolerance for the peptides in the searches was 0.6 Da for the MS spectra and 0.4 Da for the MS/MS spectra. Peptides were identified when the scoring value exceeded the identity or the extensive homology threshold value calculated by the MASCOT (*p* < 0.05).

### Bioinformatics analysis

To determine the differences among the global protein profiles of the three *Leishmania* species and to analyze the quality of the replicates, the principal component analysis (PCA) was performed using the prcomp package from the R platform [24]. A heatmap was also generated using the gplots and heatmap2 package implemented in the R platform for detecting the differences in protein profiles of the three species. For this, the fold change of the proteins with a differential abundance was initially calculated for all species. The comparison was performed using the ratio of the values of two species of *Leishmania* with respect to the third species, named denominator. The fold changes were normalized using the Z score methodology [25].

To correct any redundancy of the annotations, the BLASTp tool (https://blast.ncbi.nlm.nih.gov/Blast.cgi) was used to locate the similarity regions between the experimental and the deposited sequences in the database. The statistical significance of the comparisons was calculated, considering the results of coverage higher than 95% and identity higher than 95% as significant. After that, the correct protein NCBI ID list was obtained, and we used this list for our study. In addition, ID list from other databases such as the Uniprot and the TriTrypDB [26] was also used whenever necessary.

To evaluate the potential relationships between the differentially abundant proteins and the biological processes of the parasites, the protein-protein interaction (PPI) networks were predicted by input of the protein ID number in the STRING database version 11.0 (www.string-db.org) [27]. In Basic Settings, a cut-off score of 0.4 was used to identify interaction and the “Textmining” interaction source was deactivated. In addition, the “maximum number of interactors to show” was adjusted to “no more than 10 interactors”. The predicted networks were imported, edited, and analyzed in the program Cytoscape version 3.3.4 [28].

As STRING does not have a complete *L*. *amazonensis* and *L*. *braziliensis* database, another BLASTp was performed to convert the original ID to a similar/close species ID. The *L*. *amazonensis* ID proteins were converted to *L*. *major*, while *L*. *braziliensis* ID proteins were converted to *L*. *panamensis*. These conversions were performed only for submission to the STRING database; BLASTp was considered only for those proteins that showed ≥ 95% similarity and ≥ 95% coverage.

The Gene ontology (GO) [29] functional enrichment analysis was performed using the TriTrypDB (http://tritrypdb.org/tritrypdb/). The parameters for ontology analysis were biological processes selected; evidence code was computed and curated; the GO slim terms were limited and the p-value cutoff was 0.05. The redundant GO terms were summarized in clusters using the REVIGO tool (http://revigo.irb.hr/) [30].

### Western blotting

For fractionation of 30 μg of the promastigote protein extract from each *Leishmania* species at the logarithmic phase, SDS-PAGE 12% gel was performed. The protein bands were transferred onto nitrocellulose membranes (Hybond, Amershan, UK) in a trans-blot semidry transfer unit (GE Healthcare) by applying a current of 1.6 mA/cm^2^ for 2 h. The membranes were rinsed with PBS–Tween 0.1% and incubated with blocking buffer (5% low-fat milk powder in PBS–Tween 0.1%) at 4°C for 1 h. The transblotted proteins were probed overnight with a rabbit polyclonal anti-arginase antibody. Then, the membrane was washed with PBS–Tween 0.5% for 5 min thrice, and incubated with horseradish peroxidase (HRP)–conjugated secondary antibodies (1:1000 diluted). Specific binding was revealed with a western blotting detection ECL system (Amersham, UK) and exposed to LAS 500 (GE Healthcare, Life Sciences). The band intensities were quantified using the ImageJ 1.41 software (NIH, USA).

The gel loading control was determined by the number of parasites subjected to protein extraction, dosage of the protein extract, and application of the same amount of proteins in each well, and also by the quantification of the SDS-PAGE bands stained by Coomassie Brilliant Blue G250 [31].

### Arginase activity

Arginase activity was measured using 10^8^ logarithmic phase-promastigotes of each *Leishmania* species. Arginase activity was determined by measuring the conversion of L-arginine to L-ornithine and urea [31–34]. Promastigotes were lysed by gently shaking in 50 μL of 0.1% Triton X-100 for 30 min at room temperature. Then, 50 μL of 10 mM MnCl_2_ and 50 μL of 50 mM Tris-HCl pH 7.5 were added, followed by incubation at 55°C for 10 min. After that, 50 μL of the supernatant was mixed with 50 μL of 0.5 mM L-arginine pH 9.7 and incubated at 37°C for 1 h. Finally, the reaction was stopped with 400 μL of the stop solution containing H_2_SO_4_ (96%), H_3_PO_4_ (85%), and H_2_O in the raio of 1:3:7 (v/v/v). Then, 20 μL of α-isonitrosopropiophenone (ISPF, Sigma) dissolved in 100% ethanol were added and the mixture was warmed to 95°C for 45 min to determine the concentration of urea. Two fold serial dilutions of 1 mg/mL urea solution were employed for standard curve construction. Urea concentration was monitored at 540 nm using a spectrophotometer (Multiskan GO, Thermo Scientific), and the arginase activity unit was defined as the amount of enzyme that catalyzed the formation of 1 μmol/min of urea. These experiments were performed twice in triplicate.

Statistical analyses were performed using GraphPad Prism version 5.00 for Windows (GraphPad Software, San Diego, CA, USA). Data have been represented as mean of the group. Comparisons between different groups were made using one-way ANOVA followed by the Bonferroni’s test and *p* < 0.05 was considered significant.

## Results

### DIGE

**Fig 1:**
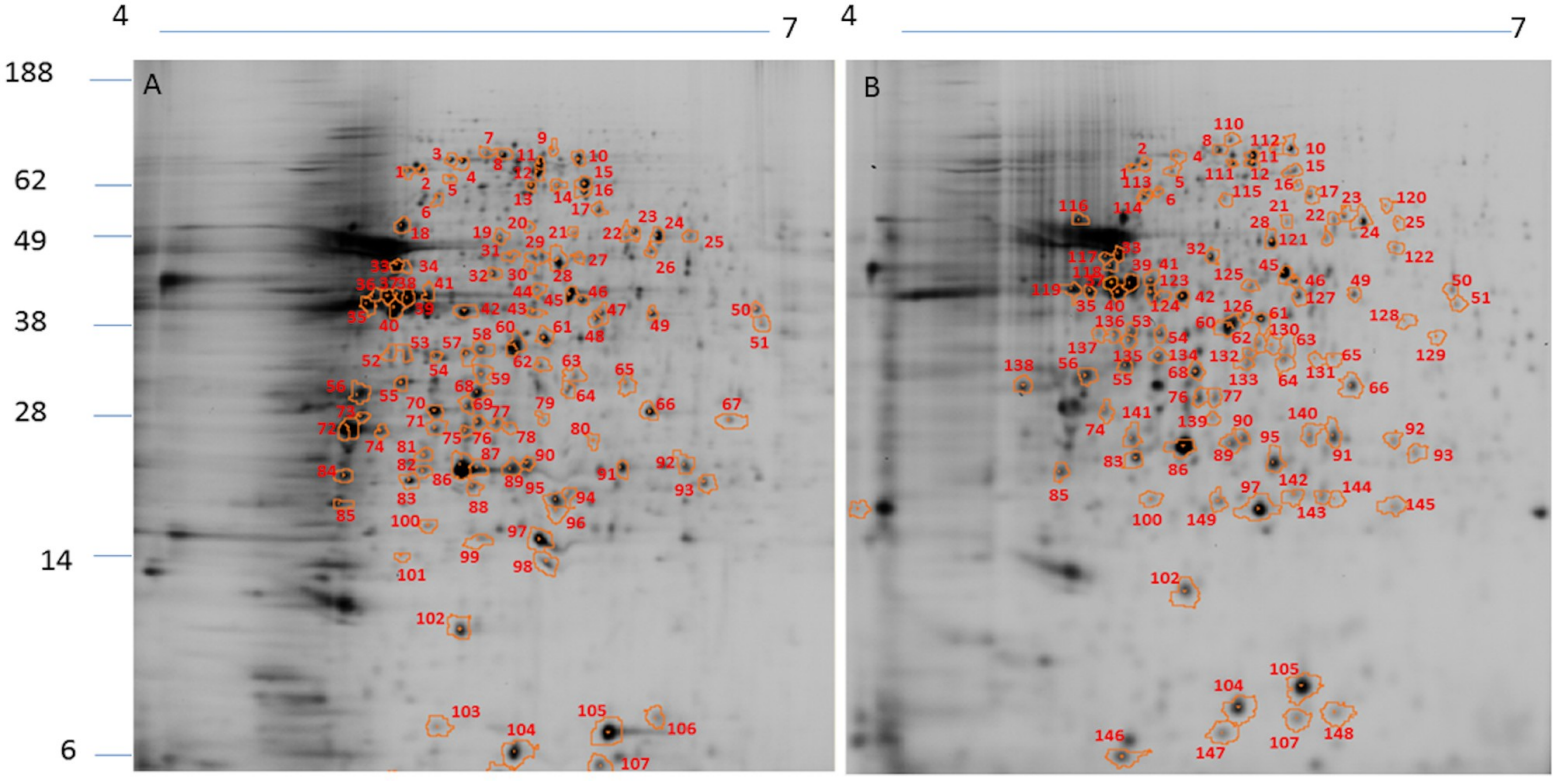
2D DIGE– 12% SDS-PAGE using IPG strips (18 cm, pH 4–7) of protein extracts from *L*. *amazonensis*. Note the spots showing higher intensity in *L*. *amazonensis* compared to those in *L*. *braziliensis* (A) and *L*. *infantum* (B). The numbers correspond to the proteins identified in S1 Table. Molecular weights (MW) have been expressed in kDa.

**Fig 2:**
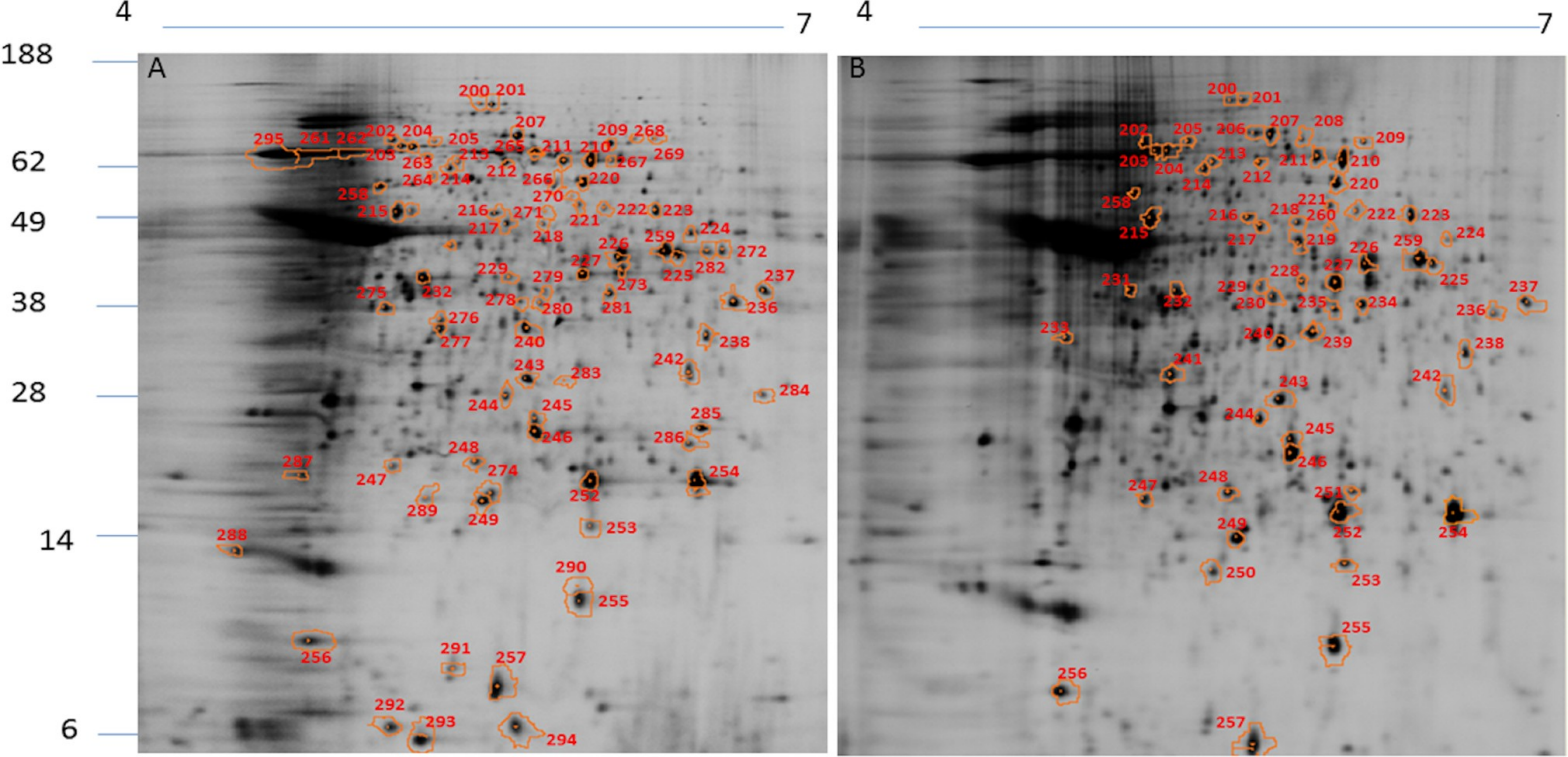
2D DIGE– 12% SDS-PAGE using IPG strips (18 cm, pH 4–7) of protein extracts from *L*. *braziliensis*. Note the spots showing higher intensity in *L*. *braziliensis* compared to those in *L*. *amazonensis* (A) and *L*. *infantum* (B). The numbers correspond to the proteins identified in S1 Table. Molecular weights (MW) have been expressed in kDa.

**Fig 3:**
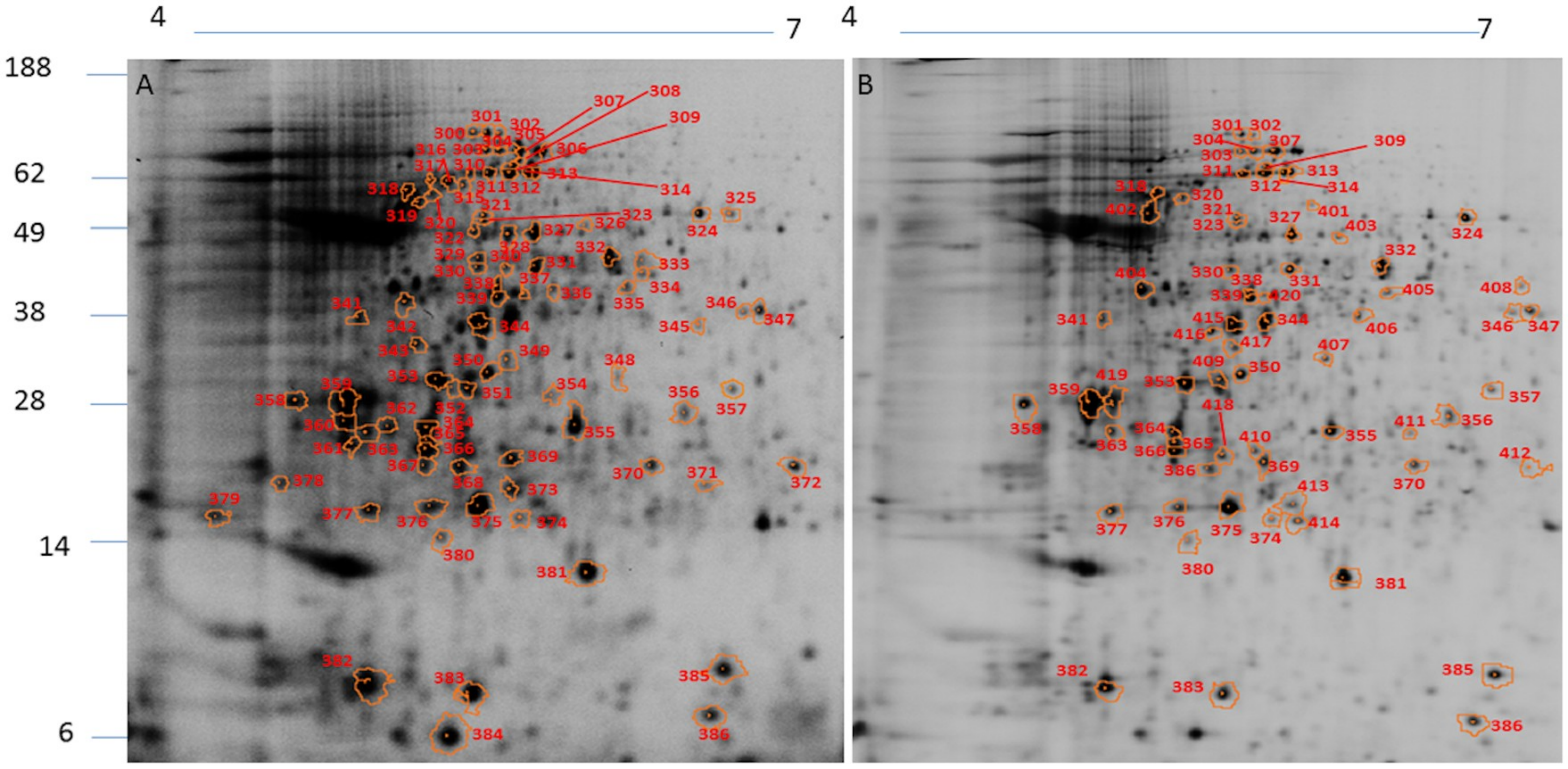
2D DIGE– 12% SDS-PAGE, IPG strips (18 cm, pH 4–7) of protein extracts from *L*. *infantum*. Note the spots showing higher intensity in *L*. *infantum* compared to those in *L*. *amazonensis* (A) and *L*. *braziliensis* (B). The numbers correspond to the proteins identified in S1 Table. Molecular weights (MW) have been expressed in kDa.

The protein extracts from *L*. *amazonensis*, *L*. *braziliensis*, and *L*. *infantum* were used for DIGE analysis. The spot profiles obtained were highly reproducible (coefficient of variation ≤ 10%) in terms of the total number of spots and their relative positions and intensities. In *L*. *amazonensis*, DIGE revealed 139 diferentially intense spots out of a total of 1154 spots (12.0%), of which 102 spots (73.4%) showed higher intensity than that in *L*. *braziliensis* and 98 (70.5%) showed higher intensity than that in *L*. *infantum* (Fig 1). In *L*. *braziliensis*, 95 out of the 1095 (8.7%) spots showed higher intensity as compared to the other two species, of which 83 (87.4%) were more intense than those in *L*. *amazonensis* and 61 (64.2%) were more intense those that in *L*. *infantum* (Fig 2). In *L*. *infantum*, 104 out of the 1216 spots (8.5%) showed higher intensity, of which 85 (81.7%) were more intense than those in *L*. *amazonensis* and 68 (65.4%) were more intense than those in *L*. *braziliensis* (Fig 3). All spots that showed higher intensity in each species in comparison with the other two species were selected for an MS analysis. The selected spots and proteins identified by MS have been listed in S1 Table. The peptide sequences were deposited at http://www.peptideatlas.org/PASS/PASS01523.

### Heatmaps and PCA

**Fig 4:**
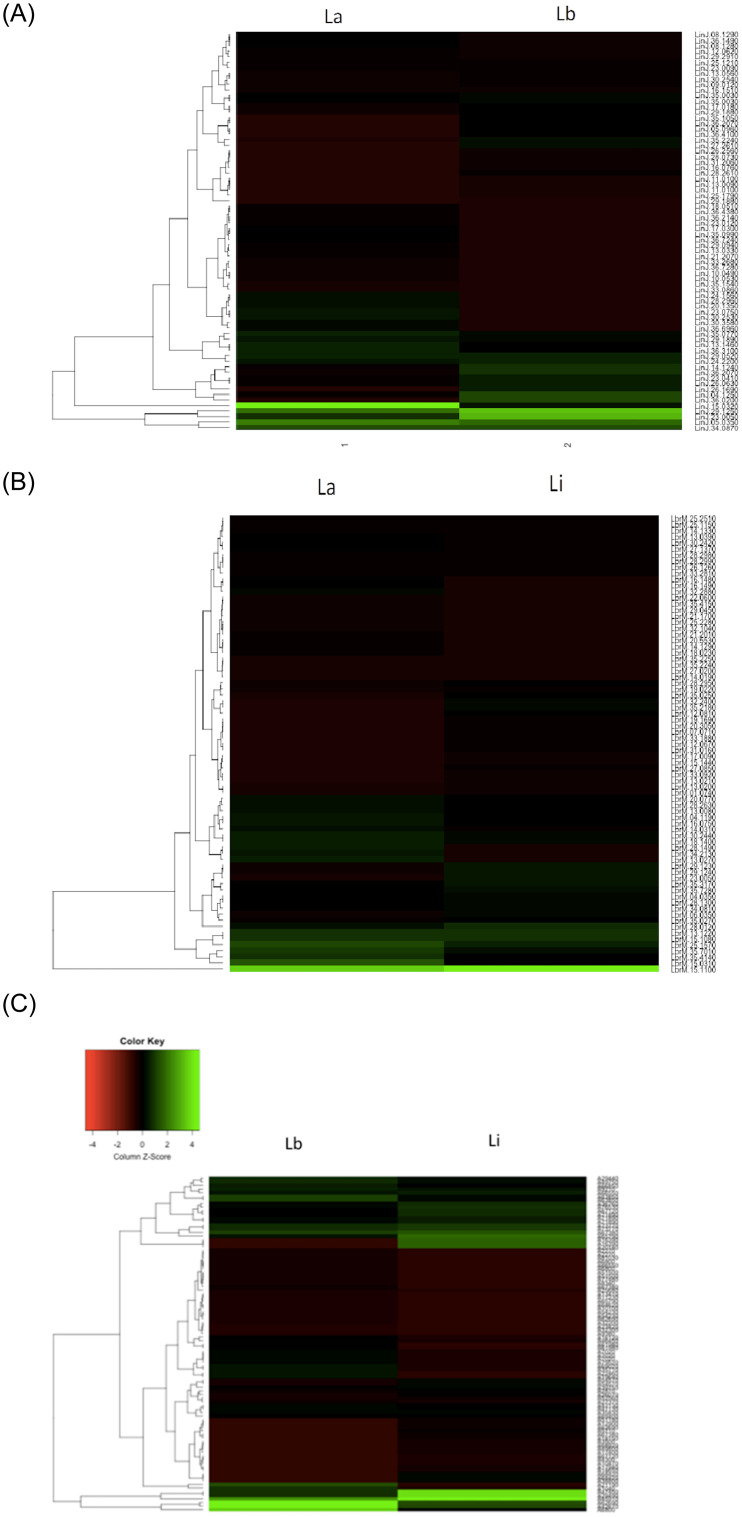
A. Heatmap of the proteins differentially abundant between *L*. *amazonensis* (La) and *L*. *braziliensis* (Lb), using the fold change of *L*. *infatum* as a denominator. B. Heatmap of the proteins differentially abundant between *L*. *amazonensis* (La) and *L*. *infantum* (Li) using the fold change of *L*. *braziliensis* as a denominator. C. Heatmap of the proteins differentially abundant between *L*. *braziliensis* (Lb) and *L*. *infantum* (Li), using the fold change of *L*. *amazonensis* as a denominator.

**Fig 5:**
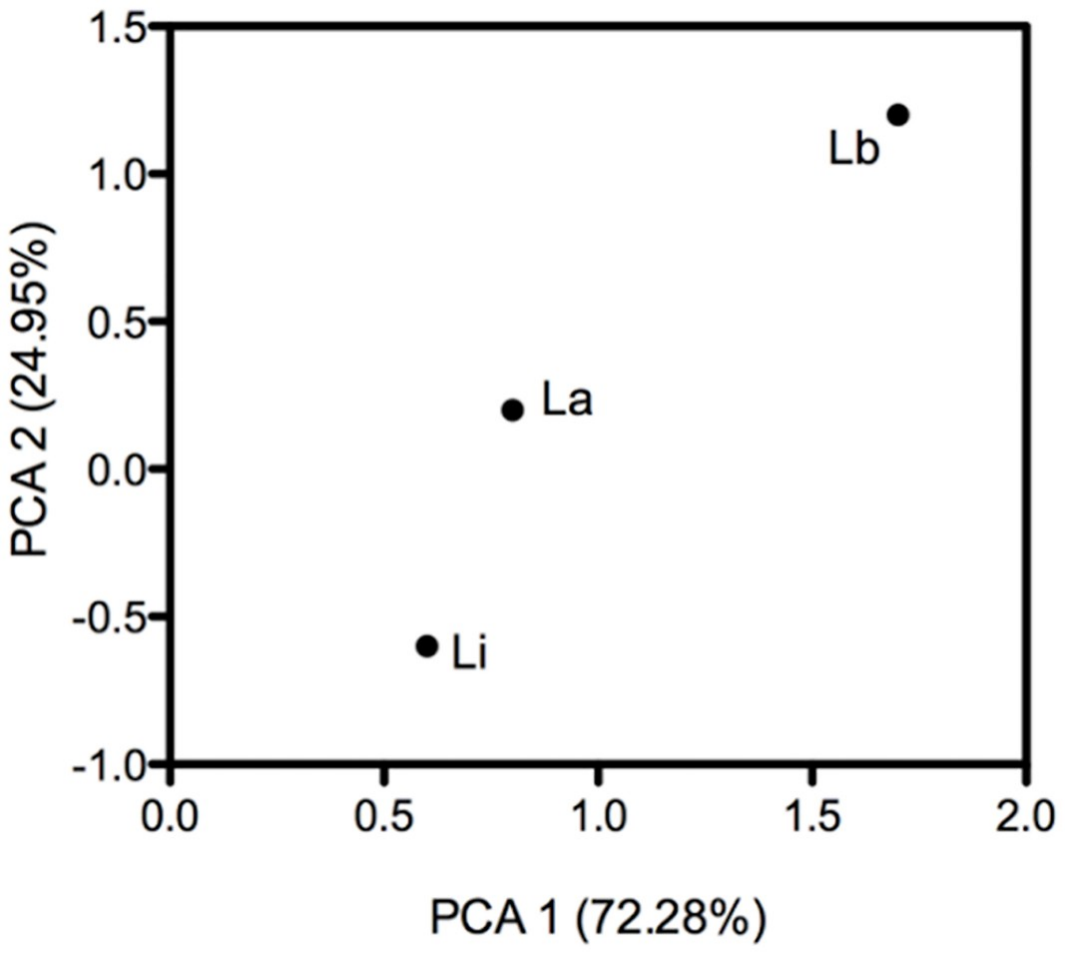
PCA analysis showing the approximation of *L*. *infantum* and *L*. *amazonensis*, and the distance from the first one in relation to *L*. *braziliensis*. The analyses considered the number and level of proteins with differences in the abundance between the species. Components 1 and 2 together represented 97.23% of the data variance.

Heatmaps were constructed to reveal the overall difference in protein levels among different *Leishmania* species, considering the fold change of each protein in the compared species in relation with the absent species used for the normalization of data. Heatmaps revealed the differences in protein abundance between *L*. *amazonensis* and *L*. *braziliensis* (Fig 4A), *L*. *amazonensis* and *L*. *infantum* (Fig 4B), and *L*. *braziliensis* and *L*. *infantum* (Fig 4C). Based on the color intensities, the difference in protein abundance between *L*. *braziliensis* and *L*. *infantum* was high, whereas the difference in protein abundance between *L*. *amazonensis* and *L*. *infantum* was low. Moreover, PCA showed that *L*. *amazonensis* and *L*. *infantum* were closer to each other, whereas both were more distant from *L*. *braziliensis*; and among the three, *L*. *amazonensis* occupied a central position (Fig 5).

### Bioinformatics analysis

One of the limitations of the gel proteomic approach is the overlapping of proteins at a spot, which may lead to an identification of more than one protein per spot. This will prevent identification of the differentially abundant proteins. Therefore, in the present work, all spots containing more than one protein were eliminated from subsequent analyses. In *L*. *amazonensis*, 43 out of the 115 spots contained more than one protein, therefore, 72 proteins were considered for analysis; in *L*. *braziliensis*, 25 out of the 77 spots contained more than one protein, therefore, 52 proteins were considered; and in *L*. *infantum*, 20 out of the 71 spots contained more than one protein, therefore, 51 proteins were considered for analysis. The proteins considered for bioinformatics analysis have been listed in S2 Table.

#### Protein-protein interactions

**Fig 6:**
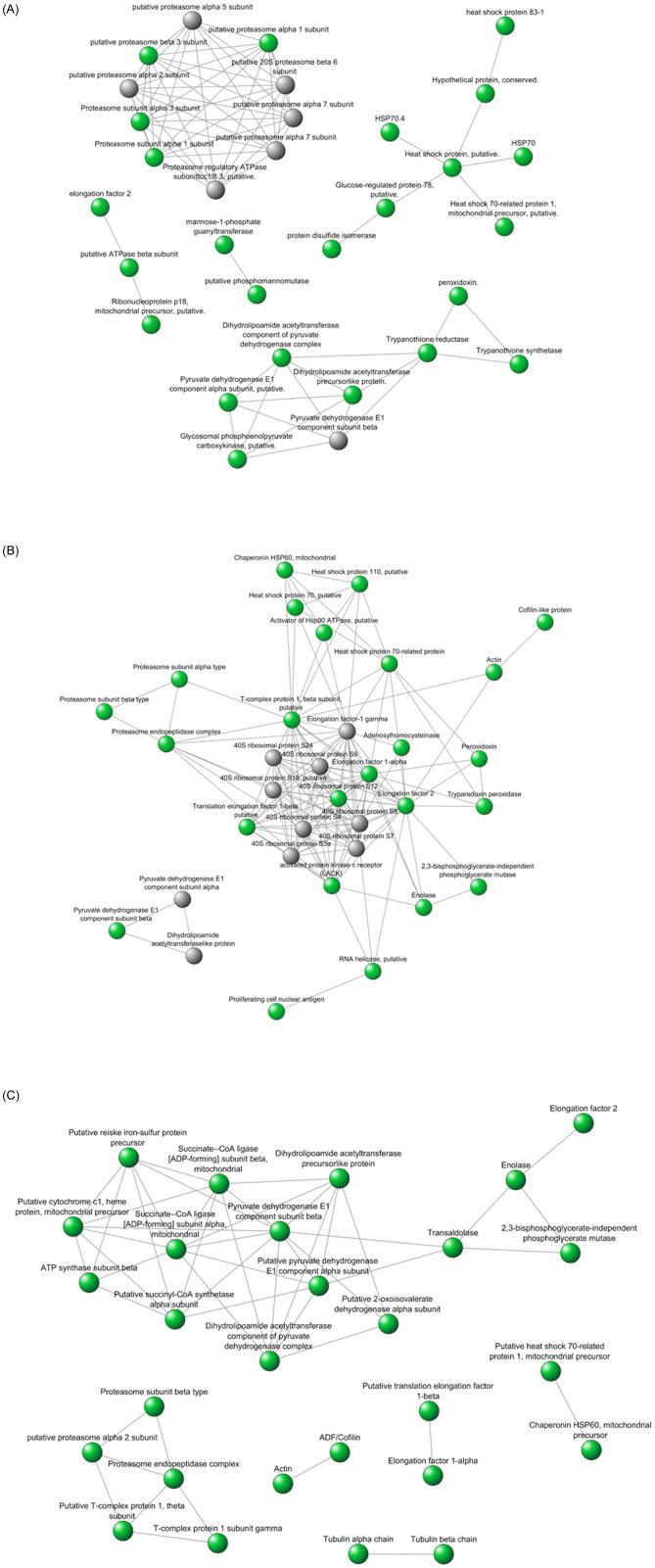
A. The interaction networks constructed on the basis of literature data and prediction available in the STRING representing an enriched subset of the differentially abundant proteins in *L*. *amazonensis* relative to *L*. *braziliensis* and *L*. *infantum*. The circles filled with green color represent the protein network involved in oxide reduction process. B. The interaction networks constructed on the basis of literature data and prediction available in the STRING representing an enriched subset of the differentially abundant proteins in *L*. *braziliensis* relative to *L*. *amazonensis* and *L*. *infantum*. The circles filled with green color represent the protein network of ribosomes. C. The interaction networks constructed with on the basis of literature data and prediction available in the STRING representing an enriched subset of the differentially abundant proteins in *L*. *infantum* relative to *L*. *amazonensis* and *L*. *braziliensis*. The circles filled with green color represent the protein network involved in energy metabolism.

Protein-protein interaction networks were constructed to evaluate the biological processes associated with the differentially abundant proteins in each species, considering that only annotation does not indicate all the metabolic and signaling pathways in which a protein can participate. Prediction of the interaction networks among the most abundant proteins in *L*. *amazonensis* (Fig 6A), *L*. *braziliensis* (Fig 6B), and *L*. *infantum* (Fig 6C) revealed that many of these proteins interacted with each other, suggesting that despite the similarity of the genome sequence, specific metabolic or signaling pathways could be differentially regulated at the protein level. The interaction networks represented an enriched subset of differentially abundant proteins from networks of *Leishmania* species previously constructed based on the literature data and prediction available in STRING. Proteins have been represented by circles, and the possibility of physical interactions between two proteins has been represented by an arrow connecting two circles; circles representing differentially abundant proteins have been filled with green color. The prominent proteins included the heat shock proteins and the protein network involved in oxide reduction process in *L*. *amazonensis*, the protein network of ribosomes in *L*. *braziliensis*, and the proteins involved in energy metabolism in *L*. *infantum* (Fig 6A, 6B and 6C).

#### Enrichment analysis

As described above, in *L*. *amazonensis* sample were enriched 72 proteins according to GO anrichment analysis, in the same way, in *L*. *braziliensis* were 52 proteins, and in *L*. *infantum* were 51 proteins (S2 Table). Six functional GO categories of biological processes were enriched in the protein set identified for each species studied: small molecule metabolic process, catabolic process, homeostatic process, protein folding, generation of precursor metabolites and energy, and nucleobase-containing compound catabolic process, the last two categories were enriched in *L*. *braziliensis* and *L*. *infantum* but not in *L*. *amazonensis*.

The differentially abundant proteins associated with each category of enriched biological processes have been described in Table 1 (*L*. *amazonensis*), Table 2 (*L*. *braziliensis*), and Table 3 (*L*. *infantum)*. It was observed that 14 proteins in *L*. *amazonensis* and *L*. *infantum*, and 11 proteins in *L*. *braziliensis* represented such categories. For the three *Leishmania* species, a total of 27 non-redundant proteins were enriched and three of them (2,3-bisphosphoglycerate-independent phosphoglycerate mutase, tryparedoxin1, and enolase), due to their diversity of function, were classified into more than one category.

**Table 1:** Proteins associated with biological processes enriched in *L*. *amazonensis*.

Biological Process	Proteins	Gene ID	Spots#	MW		pI	
				Theoric	Exp	Theoric	Exp
**Small molecule metabolic process**	**ATP synthase subunit beta, mitochondrial, putative**	**LmjF.25.1170**	18	**56,349**	62,01	**5.14**	5.1
			**34**		**55,65**		**5.1**
	**UDP-glucose pyrophosphorylase**	**LmjF.18.0990**	**17**	**54,536**	**54,3**	**5.58**	**5.8**
	**GDP-mannose pyrophosphorylase**	**LmjF.23.0110**	**126**	**41,770**	**41,7**	**5.66**	**5.7**
	Phosphoenolpyruvate carboxykinase [ATP], glycosomal	LmjF.27.1805	66	58,194	30,05	8.23	6.1
			139		28,42		5.5
	**Arginase**	**LmjF.35.1480**	**51**	**36,132**	**36,12**	**6.32**	**6.3**
	Phosphomannomutase, putative	LmjF.36.1960	88	28,101	20,03	5.18	5.5
			100		18,10		5.2
	2,3-bisphosphoglycerate-independent phosphoglycerate mutase	LmjF.36.6650	80	60,713	35,23	5.26	5.9
**Catabolic process**	**Proteasome alpha 3 subunit, putative**	**LmjF.14.0310**	**76**	**32,139**	**32,10**	**5.32**	**5.4**
	Proteasome beta 3 subunit, putative	LmjF.28.0110	143	22,463	22,01	5.32	5.8
	Tryparedoxin 1, putative	LmjF.29.1160	104	16,544	6,01	5.02	5.6
	**Proteasome alpha 1 subunit, putative**	**LmjF.35.4850**	**92**	**27,223**	**27,4**	**6.83**	**6.2**
	2,3-bisphosphoglycerate-independent phosphoglycerate mutase	LmjF.36.6650	80	60,713	35,2	5.21	5.9
**Homeostatic process**	Protein disulfide isomerase	LmjF.36.6940	33	52,377	52,4	5.42	5.1
	**Trypanothione reductase**	**LmjF.05.0350**	**24**	**53,144**	**53,03**	**7.79**	**6.2**
			120		60,01		6.3
	Peroxidoxin	LmjF.23.0040	97	25,346	15,1	6.43	5.7
			99		15,2		5.5
			142		22,03		5.8
	Tryparedoxin 1, putative	LmjF.29.1160	104	16,544	8,2	5.02	5.6
**Protein folding**	**Heat shock 70-related protein 1, mitochondrial precursor, putative**	**LmjF.30.2470**	**11**	**71,877**	**70,12**	**5.68**	5.9
			**13**		**70,02**		5.9
			**15**		**70,00**		6.0
			**111**		**70,01**		**5.6**
	Heat shock protein 83	LmjF.33.0318	35	80,406	38,02	5.09	5.0
			40		38,02		5.1
			123		38,04		5.3

Proteins are identified by their ID number and spots # (Fig 1) in which were found; and are characterized by the theoretical and experimental MW (Molecular Weight) and pI (Isoeletric Point).

**Table 2:** Proteins associated with biological processes enriched in *L*. *braziliensis*.

*Biological Process*	*Proteins*	*Gene ID*	*Spots#*	*MW*		*pI*	
				*Theoric*	*Exp*	*Theoric*	*Exp*
**Small molecule metabolic process**	**Enolase**	**LbrM.14.1330**	**259**	**46,107**	**46,1**	**5.71**	**6.24**
			**282**				**6.43**
	**Vacuolar proton pump subunit B, putative**	**LbrM.28.2630**	**216**	**55,530**	**55,5**	**5.29**	**5.3**
	2,3-bisphosphoglycerate-independent phosphoglycerate mutase	LbrM.35.7010	266	60,959	60,9	5.54	5.7
**Catabolic process**	**Enolase**	**LbrM.14.1330**	**259**	**46,107**	**46,1**	**5.71**	**6.24**
			**282**				**6.43**
	Proteasome alpha 3 subunit, putative	LbrM.14.0310	244	32,181	28,5	5.46	5.6
	Proteasome beta 3 subunit, putative	LbrM.28.0120	247	22,556	22,5	5.09	5.2
	**Tryparedoxin 1a, putative**	**LbrM.29.1230**	**254**	**16,305**	**16,3**	**5.32**	6.5
			291		8,2		**5.4**
	**Proteasome alpha 7 subunit, putative**	**LbrM.27.0200**	**286**	**25,475**	**25,3**	**5.98**	**6.3**
	2,3-bisphosphoglycerate-independent phosphoglycerate mutase	LbrM.35.7010	266	60,959	60,8	5.54	5.8
**Homeostatic process**	**Peroxiredoxin PRX1A**	**LbrM.15.1080**	**249**	**22,503**	**22,01**	**5.81**	**5.5**
			**274**		**22,51**		
	**Tryparedoxin 1a, putative**	**LbrM.29.1230**	**254**	**16,305**	**16,3**	**5.32**	6.5
			291		8,2		**5.4**
**Protein folding**	Heat shock 70-related protein 1, mitochondrial precursor, putative	LbrM.30.2420	200	70,529	140,9	5.9	5.5
			201		140,9		5.55
			209		90,5		6.0
			210		70,5		5.9
	T-complex protein 1, beta subunit, putative	LbrM.27.1370	222	57,739	57,7	5.62	6.0
	Chaperonin HSP60, mitochondrial precursor	LbrM.35.2250	263	59,513	70,02	5.34	5.3
			264		62,1		5.32
**Generation of metabolites and energy precursor AND Nucleobase-containing compound catabolic process**	**Enolase**	**LbrM.14.1330**	**259**	**46,107**	**46,1**	**5.71**	**6.24**
			**282**				**6.43**
	2,3-bisphosphoglycerate-independent phosphoglycerate mutase	LbrM.35.7010	266	60,959	60,8	5.54	5.8

Proteins are identified by their ID number and spots# (Fig 2) in which were found; and are characterized by the theoretical and experimental MW (Molecular Weight) and pI (Isoeletric Point).

**Table 3:** Proteins associated with biological processes enriched in *L*. *infantum*.

***Biological Process***	***Proteins***	***Gene ID***	***Spots#***	***MW***		***pI***	
				*Theoric*	*Exp*	*Theoric*	*Exp*
**Small molecule metabolic process**	Enolase	LINF_140018000	327	46,037	50,05	5.33	5.7
	**Transaldolase–putative**	**LINF_160013000**	**406**	**36,972**	**36,9**	**5.55**	**6.0**
	ATP synthase subunit beta—mitochondrial—putative	LINF_250018000	405	56,293	45,2	5.14	6.1
	**Inosine-adenosine-guanosine-nucleosidehydrolase—putative**	**LINF_290035800**	**341**	**36,532**	**36,5**	**4.86**	**4.8**
	Phosphomannomutase—putative	LINF_360026300	410	28,142	21,02	5.37	5.6
	**Succinyl-CoA ligase [GDP-forming] beta-chain—putative**	**LINF_360037500**	364	**44,070**	26,2	**6.77**	5.3
			**408**		**44,0**		**6.7**
	2 -3-bisphosphoglycerate-independent phosphoglycerate mutase	LINF_360078300	310	60,760	60,6	5.26	5.3
**Catabolic process**	Enolase	LINF_140018000	327	46,037	50,05	5.33	5.7
	**Proteasome alpha 2 subunit—putative**	**LINF_210026800**	**373**	**25,073**	**25,1**	**5.43**	**5.4**
	Tryparedoxin 1 –putative	LINF_290017500	383	16,697	10,02	5.2	5.5
	2 -3-bisphosphoglycerate-independent phosphoglycerate mutase	LINF_360078300	310	60,760	60,6	5.26	5.3
**Homeostatic process**	**Peroxidoxin**	**LINF_230005400**	**375**	**25,370**	**25,3**	**6.43**	**5.5**
			**413**		**25,3**		**5.7**
	Tryparedoxin 1 –putative	LINF_290017500	383	16,697	8,3	5.2	5.5
	protein disulfide isomerase 2	LINF_360081500	322	52,344	52,3	5.42	5.5
			328				5.6
**Protein folding**	Heat shock 70-related protein 1—mitochondrial precursor—putative	LINF_300030100	306	71,658	142,1	5.76	5.68
			313		80,02		5.5.6
			314		80,02		5.58
			385		10,23		6.4
	Chaperonin HSP60—mitochondrial precursor	LINF_360027200	308	59,358	140,	5.33	5.7
			311		80,02		5.5
			312		80,03		5.6
			318		70,3		5.2
			317		75,03		5.3
	T-complex protein 1—theta subunit—putative	LINF_360081100	316	57,739	57,7	5.62	5.3
**Generation of metabolites and energy precursor**	Enolase	LINF_140018000	327	46,037	50,05	5.33	5.7
	Rieske iron-sulfur protein—mitochondrial precursor—putative	LINF_350020400	349	33,726	37,9	5.91	5.6
	2 -3-bisphosphoglycerate-independent phosphoglycerate mutase	LINF_360078300	310	60,760	60,6	5.26	5.3
	**Succinyl-CoA ligase [GDP-forming] beta-chain—putative**	**LINF_360037500**	364	44,070	26,2	6.77	5.3
			408		44,0		6.7
**Nucleobase-containing compound catabolic process**	Enolase	LINF_140018000	327	46,037	50,05	5.33	5.7
	2 -3-bisphosphoglycerate-independent phosphoglycerate mutase	LINF_360078300	310	60,760	60,6	5.26	5.3

Proteins are identified by their ID number and spots# (Fig 3) in which were found; and are characterized by the theoretical and experimental MW (Molecular Weight) and pI (Isoeletric Point).

Noteworthy, although spots were selected based on the difference in their intensities in the gels of each species, only 55.5% (15 out of the 27 proteins) were unique, whereas 44.4% (12 out of the 27 proteins) were present in two or more species. This overlap occurred as a result of poin isoeletric (PI) and/or molecular weight (MW) variations of the selected spots containing the protein and, consequently, of the proteins as well. For example, the tryparedoxin protein, identified in the three investigated species, has a theoretical MW of approximately 16.5 kDa and a theoretical PI of 5.2. However, in *L*. *amazonensis* it was identified in spot #104 with 6.01 kDa MW and 5.6 PI; in *L*. *braziliensis*, it was identified in spot #254 with 16.3 kDa MW and 6.5 PI and in spot #291 with 8.2 kDa MW 5.4 PI; and in *L*. *infantum*, it was identified in spot #383 with 8.3 kDa MW and 5.5 PI. For proteins that presented different experimental and theoretical MW and PI, the functions could not be reliably discussed based on the databases because these proteins had undergone alterations, probably post-transcriptional alterations, which altered their functions. Notably, all proteins identified in more than one species showed differences between theoretical and experimental MW and/or PI, and were disregarded for discussion of their functions.

Proteins unique to each species and having experimental MW and PI similar to the theoretical ones were considered for discussion. We are calling "unique" proteins that were more abundant in only one of the species studied, we know that they are common proteins in this genus, and therefore, are not exclusive to any species. We identified eight such proteins. For *L*. *amazonensis*: arginase, ATP synthase subunit beta mitochondrial, GDP-mannose pyrophosphorylase, HSP-70 related protein 1 mitochondrial precursor, proteasome alpha 1 subunit, proteasome alpha 3 subunit, trypanothione reductase, and UDP-glucose pyrophosphate; five proteins for *L*. *braziliensis*: enolase, peroxiredoxin, proteasome alpha 7 subunit, tryparedoxin1, and vacuolar proton pump subunit B; and five proteins for *L*. *infantum*: inosine adenosine guanosine nucleosidehydrolase, peroxidoxin, proteasome alpha 2 subunit, succinyl-CoA ligase [GDP-forming] beta-chain, and transaldolase. Almost all these proteins were also found in PPI networks, suggesting a prominent role in regulating protein-protein interactions and consequently biological processes.

### Differential arginase abundance and activity

**Fig 7:**
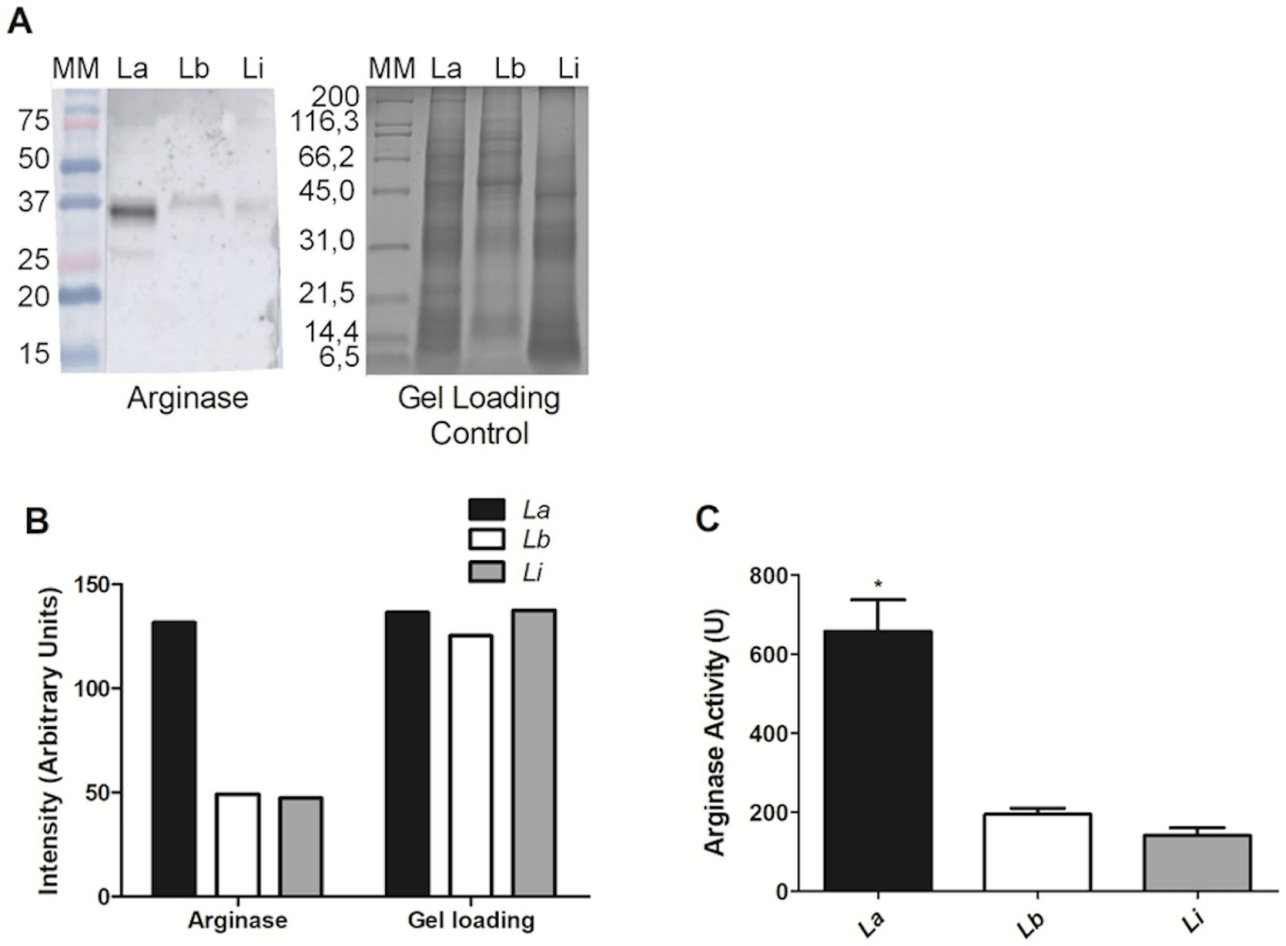
Validation of the proteomic analysis and arginase activity in *Leishmania* promastigotes. (A) Comparative western blot of *L*. *amazonensis* (L. a), *L*. *braziliensis* (L. b), and *L*. *infantum* (L. i) using anti-arginase antibody and the control. (B) Intensity measurement of the bands, in arbitrary units, by the ImageJ software (Wayne Rasband, NIH, USA, http://rsb.info.nih.gov/ij/). (C) Arginase activity in the logarithmic phase-promastigotes. These experiments were performed twice in triplicate. The arginase activity unit (U) was defined as the amount of enzyme that catalyzed the formation of 1 μmol/min of urea. *corresponds to *p* < 0.05 by one-way ANOVA.

Proteomic analysis revealed arginase to be one of the differentially abundant proteins among the three species, with higher abundance in *L*. *amazonensis*. To confirm this, we performed immunoblotting using an anti-arginase antibody which revealed a signal at the same molecular mass for all species; the signals were stronger in *L*. *amazonensis* than in other species (Fig 7A). The immunoblotting semiquantitative data as obtained by the ImageJ software has been shown in Fig 7B, which confirmed the visual evaluation of the immunoblotting signals in Fig 7A. The ratio of the signals between the strains was 2.78, 1.04, and 1.0 arbitrary units for *L*. *amazonensis*, *L*. *braziliensis*, and *L*. *infantum*, respectively. These data indicate that arginase is upregulated in *L*. *amazonensis*, corroborating the results of our proteomic analysis.

Next, we evaluated the arginase activity in *L*. *amazonensis*, *L*. *braziliensis*, and *L*. *infantum* using logarithmic phase-promastigotes. As shown in Fig 7C, arginase activity in *L*. *amazonensis* was three fold higher than that in other species (*p* < 0.05). The average activity values were 657 U, 193.8 U, and 139 U for *L*. *amazonensis*, *L*. *braziliensis*, and *L*. *infantum*, respectively.

## Discussion

Using integrated genomic and proteomic analyses, it is possible to identify the genes and proteins that can function as biomarkers for diagnosis of leishmaniasis, identify potential targets for treatment, study the establishment and progression of the disease, as well as enhance the understanding of parasite biology.

The main objective of this study was to contribute to the knowledge about the differences among *Leishmania* species by identifying the differentially abundant proteins in them. In our study, we used only one strain of each investigated species and we suggest that our data may be representative of each species; however, differences can also occur between strains of the same species. For example, PCA, which represents the global difference between the samples, shows that *L*. *amazonensis* and *L*. *infantum* are closer to each other and distant from *L*. *braziliensis*; this finding is in agreement with the phylogenetics of these species [35]. In addition, *L*. *amazonensis* occupies the central position in PCA, corroborating the fact that it causes a cutaneous lesihmaniasis like that caused by *L*. *braziliensis* and has visceral potential similar to that of *L*. *infantum*.

One limitation of the 2-DE proteomic approach was protein overlapping at the same spot, which led to an uncertainty regarding the protein of interest; therefore, spots containing more than one protein were excluded from the study. The total number of spots identified was initially 115 for *L*. *amazonensis*, 77 for *L*. *braziliensis*, and 71 for *L*. *infantum*. However, after excluding spots that contained more than one protein, the numbers of spots were 72, 52, and 51 for *L*. *amazonensis*, *L*. *braziliensis*, and *L*. *infantum*, respectively. In addition, we also observed that the mass and charge of several proteins were different from those predicted by the leishmanial genome, which has been reported to be a common feature of most proteomic analyses, probably reflecting the effect of protein maturation events including co- or post-translational modifications [36]. The database information was based on native proteins with theoretical MW and PI values, hence, the functions of these modified proteins could not be deduced from the databases. Further studies on these modifications will be required to understand the function of these proteins.

In the present study, we focused on the identification of the differentially abundant proteins among three *Leishmania* species. We began with the identification of over 100 proteins selected by DIGE; however, after enrichment analysis and exclusion of proteins with experimental MW and PI different from the theoretical values, we narrowed down to 18 proteins that could be involved in biological differences in these species. The prominent proteins included heat shock proteins and the protein network involved in oxide reduction process in *L*. *amazonensis*, the protein network of ribosomes in *L*. *braziliensis*, and the proteins involved in energy metabolism in *L*. *infantum*. According to the PPI network results, enrichment categories, and exclusive proteins analysis, the important proteins were arginase, HSPs, and trypanothione reductase in *L*. *amazonensis*; enolase, peroxidoxin, and tryparedoxin 1 in *L*. *braziliensis*; and succinyl-CoA ligase [GDP -forming] beta-chain and transaldolase in *L*. *infantum*.

For validation of the proteomic data, we evaluated the difference in arginase abundance among all three species by western blotting. It was observed that arginase was 2.6 times more abundant and showed higher activity in *L*. *amazonensis* than that in *L*. *braziliensis* and *L*. *infantum*, corroborating the results of proteomic analysis. Some researches have reported increased arginine metabolism associated with an increased arginase activity, leading to an increase in the availability of polyamines which favors the replication of the parasites [37]. The role of polyamines in parasite replication was studied using an arginase-deficient *L*. *major*; these parasites required nutritional supply of polyamines or L-eritin for their growth [38]. Polyamines are involved in the regulation of macrophage oxidative response through competition of arginine with iNOS. This competition may favor the progression of the disease and lead to a high multiplication rate of the parasites in macrophages infected by *L*. *amazonensis*. In addition, arginine provides nutrition to intracellular parasites and is also involved in the parasite’s replication in insects and mammals [37, 39–41]. *L*. *amazonensis* promastigotes showed increased arginase activity than those exhibited by amastigotes [42], which corroborates the hypothesis called pre-adaptation, supported by other research [43–45], which states that this preparation is essential for survival of the parasite in the host cell phagolysosome. Arginase is essential for infectivity, proliferation, and virulence of the parasite [46, 47], and the finding that it is increased in *L*. *amazonensis* relative to *L*. *braziliensis* and *L*. *infantum* will help us understand the reason of considerable increase in the counts of the parasite in *L*. *amazonensis* infections as compared to the infections by the other two species.

HSPs participate in a large number of biochemical and immunological pathways. They behave as chaperones, as immunodominant antigens, and are also implicated in the antigen-processing pathway [48]. When a parasite enters a mammalian host, it encounters an environment with a higher temperature, which leads to the synthesis of proteins responsible for cellular response against high temperatures and other stresses, stimulating the differentiation to an intracellular form. In this context, HSP70 plays a key role and its absence leads to a decreased replication and virulence of the parasite [49]. *Leishmania* secretes virulence factors into the host cytoplasm, where they interact with host signaling molecules to subvert the host immune responses [50]. HSP70 and enolase have been identified among the proteins present in the exoproteome of *L*. *donovani*, *L*. *mexicana*, and *L*. *braziliensis*, and are potential targets for the development of new antileishmanial drugs and/or new vaccines for leishmaniasis [51]. The presence of HSPs in exosomes most likely ensures the correct folding of exosomal proteins, consequently functioning as virulence factors. We suggest that the increased abundance of HSP70 in *L*. *amazonensis* may corroborate its ability to establish an infection at several sites including the skin and the viscera.

According to the existing literature, trypanothione reductase (TR) activity has been directly associated with the infectivity of *L*. *amazonensis*. Castro Pinto et al., 2004 evaluated TR activity based on the consumption of NADPH (nicotinamide adenine dinucleotide phosphate hydrogen); a higher consumption of NADPH and consequently higher enzyme activity was observed in infective promastigotes as compared to those in the non-infective ones [52]. Moreover, when compared with promastigotes, amastigotes showed an even higher enzymatic activity than that in the flagellate forms. In most organisms, the intracellular redox environment is maintained by glutathione reductase, however, in trypanosomatids, this enzyme is absent and TR is responsible for maintaining trypanothione in its reduced form, protecting the parasite from reactive oxygen species produced by the host [53–55].

Enolase (2-phospho-D-glycerate hydrolase, EC 4.2.1.11) is known to catalyse the reversible dehydration of D-2-phosphoglycerate (2PGA) to phosphoenolpyruvate (PEP) in glycolysis as well as gluconeogenesis, the two metabolic pathways vital for cellular function. This enzyme is generally highly conserved, with similar overall fold and identical catalytic residues in all organisms. Enolase is found in the secretome as well as in association with the surface of *Leishmania* spp., where it probably functions as a plasminogen receptor, playing a role in the parasite’s invasiveness and virulence, a function possibly present in other trypanosomatids as well. This location and possible function of enolase offer additional perspectives for both drug discovery and vaccination [56]. In fact, a recent study demonstrated the prophylactic effect of *L*. *donovani* enolase on *L*. *donovani* infected hamsters [57]. Enolase is also upregulated in promastigotes as compared to that in the amastigotes, at least in *L*. *major*, *L*. *infantum*, *L*. *donovani*, and *L*. *pifanoi* [58]. Thus, enolase has multiple functions and locations, making it very difficult to understand, or even speculate, the role of its increased abundance in *L*. *braziliensis*.

Proteins such as peroxidoxin and tryparedoxin peroxidase (TryP) interact with each other, and both are associated with virulence [59] and drug resistance of *Leishmania* [60, 61]. Peroxidoxins comprise a family of antioxidants that have been recently discovered in numerous prokaryotes and eukaryotes and play key roles in defense against oxidative stress. Both peroxidoxin and TryP are critical to the survival of *Leishmania* during oxidative stress generated by macrophages and drugs [62]. TryP participates in defense against oxidative stress by catabolism of hydrogen peroxide into water molecules [63]. In addition to cellular detoxification of reactive oxygen species, TryP has been involved in other processes such as signaling cellular proliferation and differentiation [64]. The complex expression pattern of TryP variants found in *L*. *amazonensis* promastigotes suggests that TryP is especially important throughout the growth and differentiation process [59]. Our results suggest that *L*. *braziliensis* may be more effective in this redox pathway than those observed in *L*. *amazonensis* and *L*. *infantum*. However, this hypothesis needs to be tested and it is also necessary to investigate the effect of this greater abundance on the biology of this species.

Finally, in *L*. *infantum*, several proteins involved in energy metabolism were identified to be more abundant than in the other two species, including the succinyl-CoA ligase [GDP-forming] beta-chain and transaldolase (which were exclusively abundant in this species). In *Leishmania*, very few studies have been undertaken to evaluate the role of these enzymes, and it was shown that *L*. *donovani* overexpressing transaldolase (TAL-OE) were less susceptible to oxidative stress and more resistant to sodium antimony gluconate (SAG), amphotericin B (AmB), and miltefosine because of increased availability of NADPH which maintained the intracellular redox balance perturbed by the cited drugs. Moreover, the authors showed that parasites TAL-OE prevented an oxidative stress-induced protein carbonylation and lipid peroxidation [65]. The succinyl-CoA ligase [GDP-forming] beta-chain participates in the tricarboxylic acid cycle, a nearly universal metabolic pathway in which the acetyl group of acetyl coenzyme A is effectively oxidized to two molecules of CO_2_ and four pairs of electrons are transferred to coenzymes. This enzyme is downregulated in the mid-logarithmic phase *L*. *pifanoi* promastigotes [58], but is constitutively expressed in *L*. *amazonensis* promastigotes [42]. An increase in the abundance of this enzyme was observed in the potassium antimonyl tartrate (SbIII)-susceptible lines of *L*. *braziliensis* [61], whereas the SbIII-resistant line of *L*. *infantum* showed a reduction in this protein [60]. Taken together, the proteins abundant in *L*. *infantum* favor an enhanced glycolysis, which provides energy for their proliferation and helps reduce oxidative stress. However, the role of these proteins in the greater chronicity of *L*. *infantum* infection remains unknown.

The difference in protein abundance among different *Leishmania* species is a broad field of study and can reveal several unexplored aspects of *Leishmania* biology. We believe that our study has revealed certain potential targets for diagnosis and treatment of leishmaniasis. However, additional investigations must be performed to understand several aspects of *Leishmania* species and clinical forms.

## Supporting information

S1 Raw images (PDF)

S1 Table The selected spots and proteins identified by MS/MS. (XLSX)

S2 Table Proteins considered for bioinformatics analysis. (XLSX)
