# Supplementary material for: Study of the differentially abundant proteins among Leishmania amazonensis, L. braziliensis, and L. infantum
Source: PLoS One. 2020 Oct 15;15(10):e0240612. doi: 10.1371/journal.pone.0240612 (PMC7561129; doi:10.1371/journal.pone.0240612)
Supplement: S1 Raw images — (PDF) [file pone.0240612.s001.pdf]

FIGURE 7

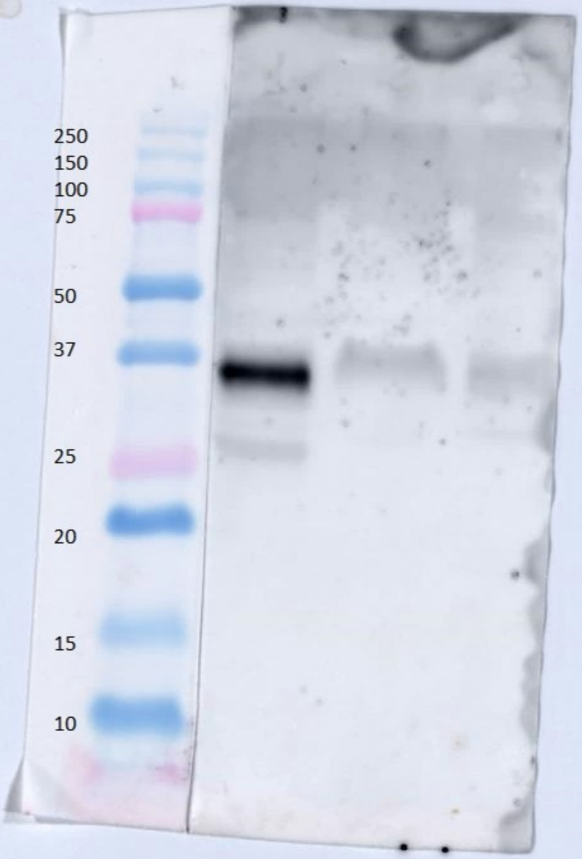

Method used to capture the image:
Electrochemiluminescence - ECL

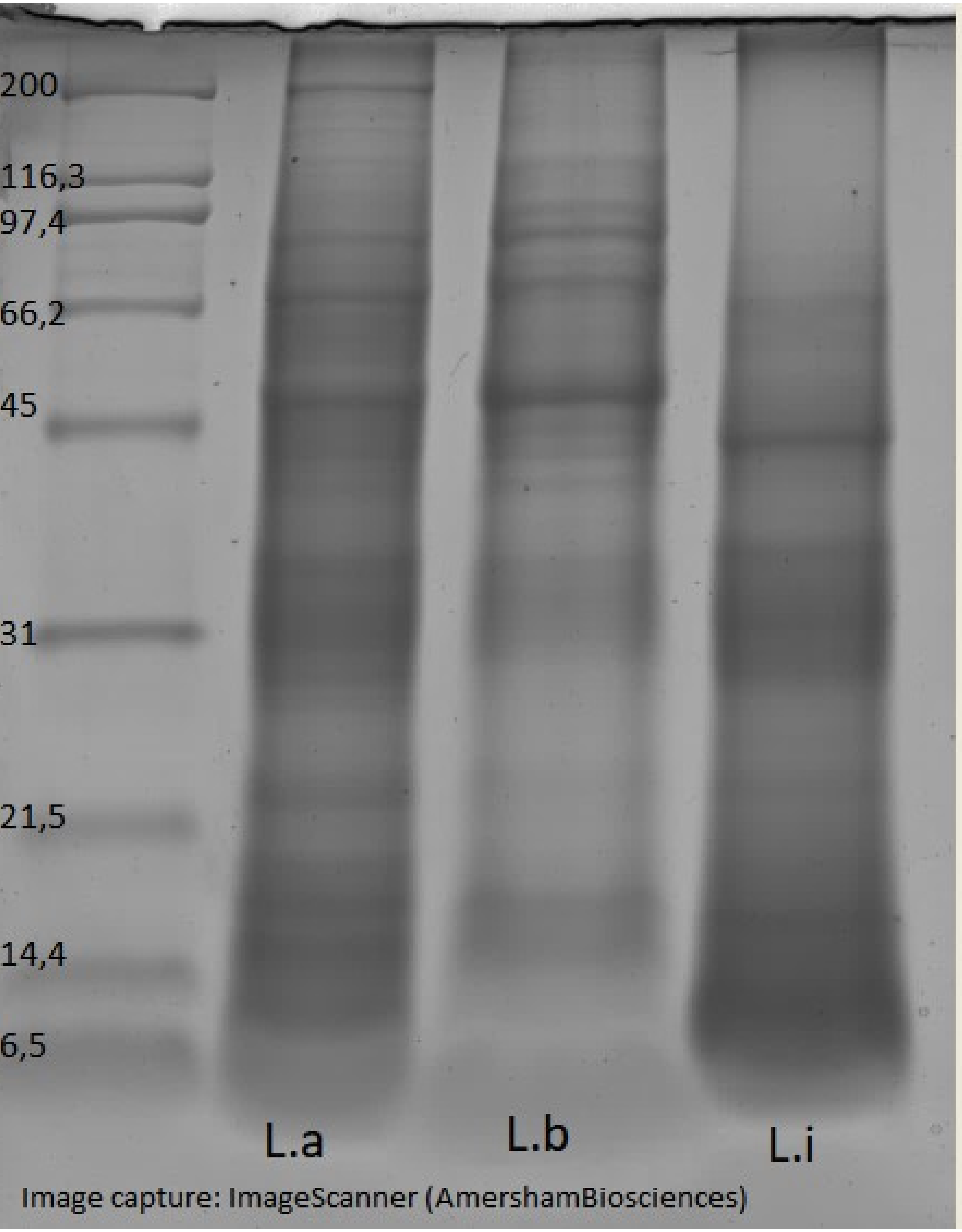
200
116,3
97,4
66,2
45
31
21,5
14,4
6,5
L.a
L.b
L.i
Image capture: ImageScanner (AmershamBiosciences)
